# Nicotinic Receptors in Sleep-Related Hypermotor Epilepsy: Pathophysiology and Pharmacology

**DOI:** 10.3390/brainsci10120907

**Published:** 2020-11-25

**Authors:** Andrea Becchetti, Laura Clara Grandi, Giulia Colombo, Simone Meneghini, Alida Amadeo

**Affiliations:** 1 Department of Biotechnology and Biosciences, University of Milano-Bicocca, 20126 Milano, Italy; laura.grandi@unimib.it (L.C.G.); g.colombo83@campus.unimib.it (G.C.); simone.meneghini@unimib.it (S.M.); 2 Department of Biosciences, University of Milano, 20133 Milano, Italy; alida.amadeo@unimi.it

**Keywords:** autosomal dominant nocturnal frontal lobe epilepsy (ADNFLE), autosomal dominant sleep-related hypermotor epilepsy (ADSHE), antiepileptic, cholinergic receptor nicotinic alpha 2 subunit (CHRNA2), cholinergic receptor nicotinic alpha 4 subunit (CHRNA4), cholinergic receptor nicotinic beta 2 subunit (CHRNB2), K^+^-Cl^−^ cotransporter type 2 (KCC2), neuronal nicotinic acetylcholine receptor (nAChR), synaptogenesis

## Abstract

Sleep-related hypermotor epilepsy (SHE) is characterized by hyperkinetic focal seizures, mainly arising in the neocortex during non-rapid eye movements (NREM) sleep. The familial form is autosomal dominant SHE (ADSHE), which can be caused by mutations in genes encoding subunits of the neuronal nicotinic acetylcholine receptor (nAChR), Na^+^-gated K^+^ channels, as well as non-channel signaling proteins, such as components of the gap activity toward rags 1 (GATOR1) macromolecular complex. The causative genes may have different roles in developing and mature brains. Under this respect, nicotinic receptors are paradigmatic, as different pathophysiological roles are exerted by distinct nAChR subunits in adult and developing brains. The widest evidence concerns α4 and β2 subunits. These participate in heteromeric nAChRs that are major modulators of excitability in mature neocortical circuits as well as regulate postnatal synaptogenesis. However, growing evidence implicates mutant α2 subunits in ADSHE, which poses interpretive difficulties as very little is known about the function of α2-containing (α2*) nAChRs in the human brain. Planning rational therapy must consider that pharmacological treatment could have different effects on synaptic maturation and adult excitability. We discuss recent attempts towards precision medicine in the mature brain and possible approaches to target developmental stages. These issues have general relevance in epilepsy treatment, as the pathogenesis of genetic epilepsies is increasingly recognized to involve developmental alterations.

## 1. Introduction

Epilepsy is a common neurological disease, whose hallmark is the presence of recurring “seizures”, i.e., transient events of abnormal neuronal activity in the brain that cause recognizable signs [1]. Overall, life expectancy is lower in epileptic patients, and the incidence of sudden death higher, not to speak of the deteriorated quality of life and social stigma [2]. The symptoms can be often controlled by antiepileptic drugs (AEDs), which can nonetheless cause serious side effects. Moreover, about 30% of the patients are refractory to pharmacological treatment. For 10% to 50% of these, depending on age and the site of seizure, surgery can be an effective therapeutic option [3].

Epileptic syndromes are heterogeneous, and so is their severity and the spectrum of cognitive, behavioral and psychiatric comorbidities. Epilepsies can be broadly classified into focal (arising in networks limited to one hemisphere), generalized (rapidly engaging both hemispheres), combined focal and generalized, and epilepsies with unknown onset [4]. The known etiological factors can be structural (e.g., following trauma), genetic, immune, infectious, or metabolic [5]. In general, research on genetic epilepsies offers invaluable suggestions about the underlying cellular and molecular mechanisms, and on how to test hypotheses about the pathogenesis as well as novel therapeutic approaches in animal models [6]. However, despite its wide diffusion in human populations (with an incidence of 3–4% in industrialized countries; [7]), epilepsy remains a relatively neglected disease [2]. Much further effort is thus needed to analyze more deeply the epileptogenic process, which will hopefully suggest how to develop new efficacious treatments to control the symptoms and, possibly, cure the disease.

## 2. Sleep-Related Hypermotor Epilepsy (SHE)

In 1977, Pedley and Guilleminault described for the first time sleepwalking in patients accompanied by screaming, complex automatisms and epileptiform electroencephalographic (EEG) events [8]. Shortly afterwards, Lugaresi and Cirignotta reported on patients showing sleep related tonic spasms and hyperkinetic movements [9]. Subsequent observations established the epileptic nature of these events and led to the term nocturnal frontal lobe epilepsy (NFLE), to denote epilepsy characterized by short seizures arising in the frontal lobe during non-rapid eye movements (NREM) sleep and accompanied by prominent motor manifestations [10,11]. Recently, the NFLE denomination was replaced by sleep-related hypermotor epilepsy (SHE) because (1) seizures are present during day- and night-sleep; (2) they can originate from frontal as well as extra-frontal areas [12,13]. In particular, SHE is characterized by brief (˂2 min) hypermotor seizures that mainly occur during stage 2 of NREM sleep and may be preceded by sudden arousals. The hypermotor events consist of complex and vigorous body movements, whose individual pattern tends to be highly stereotyped, suggesting disinhibition of subcortical motor modules. The most severe forms may be associated with cognitive deficits, intellectual disabilities and psychiatric comorbidities [12,13,14]. The first-line treatment is based on the use of carbamazepine [15], which can however cause serious side effects. As in most other epilepsies, about 30% of SHE patients are drug-resistant, and surgery can be resolutive in a fraction of these patients [16]. Contrary to previous opinion, recent evidence from a wide cohort of SHE patients indicates that the long-term outcome is rarely favorable, with only ~22% of the patients showing 5-year seizure remission [17]. Remission is affected by age of onset and the occurrence of seizures in wakefulness [17].

## 3. The Implication of Neuronal Nicotinic Receptors (nAChRs) in Autosomal Dominant SHE (ADSHE)

The incidence of SHE among epileptic patients is estimated to be approximately 1.8 per 100,000 individuals [12]. Among SHE cases, 10% to 15% have genetic background and present autosomal dominant transmission [11,18,19]. The first mutations linked to ADSHE (previously known as autosomal dominant nocturnal frontal lobe epilepsy (ADNFLE)) were found on genes encoding nAChR subunits [19]. More recently, other genes were implicated in the disease. In the following subsections, we overview the nAChR function in the neocortex and discuss the ADSHE-linked mutations relative to nAChR subunits, the main focus of the present review. Other genes linked to ADSHE are briefly described in Section 4. The pharmacological and developmental aspects are respectively discussed in Section 5 and Section 6, especially as they relate to nAChRs.

### 3.1. nAChRs in the Cerebral Cortex

In the neocortex and thalamus, acetylcholine (ACh) is mainly released by fibers ascending from cholinergic nuclei respectively located in the basal forebrain and pons [20], but intra-cortical cholinergic cells are also known [21,22]. Cholinergic nuclei are highly active during wakefulness, strongly decrease their activity during NREM sleep, and reactivate during REM sleep [20]. ACh can activate metabotropic muscarinic and ionotropic nAChRs [23]. The latter are ion channels constituted by five subunits surrounding a central pore permeable to cations. The mammalian neocortex widely expresses the α4, α7, and β2 nAChR subunits, respectively coded by *CHRNA4*, *CHRNA7*, and *CHRNB2* [24,25]. The main nAChR subtypes in the brain are the homopentamer (α7)_5_ and the heteropentamer α4β2*. The former presents lower affinity for ACh, with half-effective concentration (EC_50_) of ~100–200 µM, quick desensitization (time constant in the order of milliseconds), and a permeability ratio between Ca^2+^ and Na^+^ (P_Ca_/P_Na_) of ~10 [26,27]. Heteromeric α4β2 nAChRs have lower affinity for the agonists (with apparent EC_50_ for ACh and nicotine of ~10 µM), a much lower permeability to Ca^2+^ (P_Ca_/P_Na_ ≈ 2), and slow desensitization (time constant in the order of seconds; [26,27]). More specifically, two main stoichiometries of α4β2 nAChRs coexist: the high-affinity (α4)_2_(β2)_3_ (with EC_50_ in the low micromolar range) and the low-affinity (α4)_3_(β2)_2_ (EC_50_ ~60 µM; [28]). Other major nAChR subunits in the neocortex are α2 (encoded by *CHRNA2*), α5 (*CHRNA5*), and β4 (*CHRNB4*), which can associate with α4 and β2 to exert specific physiological roles in different neuronal populations and different developmental stages [29,30,31,32,33]. While the distribution of α4 and β2 is broadly similar in Rodents and Primates (reviewed in [31]), differences are observed in the distribution of α2 and β4 [34]. In the cerebral cortex, both subunits are much more widespread in squirrel monkeys [35] and *Macaca mulatta* [36], compared to Rodents [34]. In the latter, α2 appears to have a more localized expression, in specific GABAergic cells of deep layers [33,37,38], It is presently unclear to what extent such differences extend to the human species. Nonetheless, in those regions where it has been measured, α2 expression is higher in humans than in mice [34]. Regardless of the properties of individual subtypes, the lower affinity for ACh and the slow desensitization enable heteromeric nAChRs to regulate excitability on a wider time scale, compared to α7.

Ion currents flowing through nAChRs drive membrane potential towards the reversal potential of these channels, which results to be approximately 0 mV, irrespective of subunit composition [39]. Hence, nAChR activation generally depolarizes the cell, which can lead to activation of voltage-gated Na^+^ and Ca^2+^ channels. Moreover, Ca^2+^ influx through nAChRs can stimulate calcium-induced calcium release from intracellular stores, particularly at presynaptic sites [40]. Broadly speaking, the effect of nAChR activation across cortical layers tends to be excitatory (e.g., [41]). However, one should keep in mind that nAChRs are found on the soma and synaptic terminals of both excitatory and inhibitory neurons, with a complex developmental pattern. Therefore, the specific effects of nAChR activation on local networks in different regions, at different stages, are difficult to predict. An important and debated issue is the time-course of ACh action, which may have different impact on receptor subtypes with different kinetics and sensitivity to the agonists, as is often the case in mutant ADSHE-linked receptors. The balance of slower (paracrine) and faster (synaptic) ACh effects is a vexed question (recently discussed in [42,43]). The kinetics of ACh concentration in the smallest extracellular domains depends on a variety of factors and can be only partially analyzed with current methodologies. Nonetheless, there is agreement that the timescale of ACh action ranges from milliseconds to at least seconds. In fact, ACh release events evoked by sensory cues or optogenetic stimulus present a rise time of 0.2–0.5 s and peak levels of approximately 4–6 s [44]. Such a time scale covers the time constant of desensitization of different heteromeric nAChR subtypes, justifying the conclusion that the alterations of current kinetics and sensitivity to the agonists observed in ADSHE-linked nAChR variants (discussed later) is a relevant factor in causing pathophysiological effects in neocortex networks.

### 3.2. The α4 and β2 nAChR Subunits in ADSHE

In 1995, a missense mutation on *CHRNA4* was associated with ADSHE [45]. This landmark study provided the first evidence of a gene linked to epilepsy and pointed to mutant ion channels as a major causal factor in genetic epilepsy. ADSHE-linked mutations were soon also found on *CHRNB2* [46,47], and the number of ADSHE mutations found on *CHRNA4* or *CHRNB2* has subsequently increased at a steady pace [19,48,49]. Recently, variants of heteromeric nAChR subunits have been also suggested to be implicated in other epileptic forms [50,51,52]. In contrast, despite the wide expression of α7 receptors in the brain [30], evidence about the involvement of *CHRNA7* in epilepsy is weak. The 15q13.3 microdeletion, which comprises seven genes (*quorum CHRNA7*), has been correlated with genetic predisposition to several neurological pathologies, including idiopathic generalized epilepsy [53]. However, the specific role, if any, of *CHRNA7* copy number variation is unknown. The difficulty of clarifying this issue is exacerbated by the fact that deleting *CHRNA7* in murine models causes no evident pathologic or behavioral effects [54,55], which questions the suitability of murine models in this pathophysiological context.

Most ADSHE-linked mutations falling on *CHRNA4* or *CHRNB2* appear to cause a “gain-of-function” phenotype in classical expression systems such as *Xenopus* oocytes and human embryonic kidney cell lines [48,56], by increasing the receptor’s sensitivity to the agonists [47,57], accompanied or not by a shift of the steady-state desensitization curve [46,58]. The molecular mechanisms underlying these alterations remain somewhat controversial, especially regarding whether these mutations affect the balance of receptor’s stoichiometries, or alter the intrinsic binding capacity, or both (e.g., [58,59,60,61]). Nonetheless, these observations suggest that mutant nAChRs may be abnormally activated in conditions of low ACh release, as is typical of NREM sleep. However, the possible compensatory responses of the brain to altered nAChR function should not be neglected. In fact, a positron emission tomography study in a group of patients carrying different ADSHE mutations (on either *CHRNA4* or *CHRNB2*) showed an altered distribution of nAChRs, including a decreased density in right dorsolateral prefrontal cortex, accompanied by an increase in several subcortical regions [62].

To better understand the pathophysiology, mouse [63,64,65,66,67] and rat [68,69,70] strains have been generated that express mutant α4 or β2 subunits linked to ADSHE. Some of the mutant strains display spontaneous seizures during slow-wave sleep [68] or periods dominated by slow-wave EEG activity [65]. Other strains display phenotypic features belonging to the ADSHE semiology, such as dystonic arousal complex [64], spontaneous seizures accompanied by altered EEG pattern, but unrelated to sleep [63,69], or disturbances of the normal sleep pattern [66]. The physiological and morphological study of the effect of these mutations on neocortical neuronal populations and networks is still somewhat fragmentary, but a spectrum of alterations of GABAergic activity has been reported. Some mutations (α4^Ser280Phe^ and α4^insL^) lead to hyperactivation of inhibitory neurons [63], which could lead to synchronize pyramidal neurons, whereas α4^Ser284Leu^ decreased GABA release in rat sensorimotor cortex, which suggests network disinhibition [68], followed by upregulation of the mitogen-activated protein kinase/extracellular signal-regulated kinase pathway and expression of connexin 43 [70]. Whether different mutations indeed cause epileptogenic effects by distinct mechanisms, or a given mutation can produce different effects in different neuronal populations or brain states remains an open question. Finally, conditional expression of the ADSHE mutation β2^Val287Leu^ indicates that some of the permanent defects of the neocortical network may be produced at early postnatal stages [65,71]. This point is further discussed later.

### 3.3. The α2 nAChR Subunit and Its Mysteries

The critical implication of the cholinergic system in sleep-related epilepsy was further suggested by the identification of a mutation on *CHRNA2*, giving the non-synonymous substitution Ile279Asn, linked to a familial epilepsy with nocturnal wandering and ictal fear [72]. In this case, mutant nAChR expression in heterologous systems displayed a strong increase in the nAChR sensitivity to the agonists [72,73], similar to what was previously observed in several ADSHE-linked mutations on α4 and β2 [57]. The peculiar neurological phenotype may depend on the high expression of the α2 subunit in the habenular-interpeduncular pathway [25,74], as well as in specific neuronal groups in the hippocampus-subiculum [75,76] and amygdala [77].

Following-up the identification of α2^Ile279Asn^, several large-scale genetic studies were carried out on European patients presenting classical sporadic or familial ADSHE, finding no evidence of epilepsy-linked *CHRNA2* mutations [78,79]. More recently, in a Chinese cohort of 257 patients (42 familial and 215 sporadic cases) three *CHRNA2* single nucleotide polymorphisms have been identified, two of which lead to non-synonymous amino acid substitutions (Thr22Ile and Thr125Ala) [80]. For these, no functional studies are yet available. However, two *CHRNA2* mutations identified in Italian families affected by ADSHE were found to present a “loss-of function” phenotype, when expressed in human cell lines [81,82]. In particular, α2^Tyr252His^ strongly reduces the number of channels bound to the agonist, without significantly altering the overall channel expression [82].

The apparently opposite behavior of mutant α2-containing (α2*) and α4β2* nAChRs linked to typical ADSHE may depend on the specific functions of α2 in the human brain. The distribution of α2 in neocortex and thalamus differs between Rodents and Primates, with expression being considerably higher in the latter. This led to hypothesize that the subunit promoter was still evolving at the time of divergence between the two lineages [36]. Be that as it may, no evidence of seizures is found in mice after the deletion of α2, but rather a potentiation of several nicotine-induced behaviors [83]. Hence, designing good animal models for this form of ADSHE will not be straightforward, and determining the detailed function of α2* nAChRs in relation to human epilepsy will require substantial further investigation, especially considering that no good pharmacological tools are available to distinguish this nAChR subtype.

At the present time, we can hypothesize two possible pathogenetic mechanisms (not mutually exclusive). First, in mouse neocortex, non-desensitizing α2* nAChRs are specifically expressed in Martinotti interneurons, whose 15 Hz-bursting (β band) activity effectively synchronizes the thick-tufted pyramidal cells in layer V [33]. A defective cholinergic response in Martinotti cells could favor pyramidal cell disinhibition and hamper the alternation of UP and DOWN states during NREM sleep oscillations. Testing this hypothesis in surgical samples from the human neocortex would not be straightforward. Second, as other nicotinic subunits, α2 presents a peak of expression during the second postnatal week and is thought to be implicated in synaptogenesis [84], The specific effects of α2 at this stage are virtually unknown. It is possible that expression of a non-functional channel protein affects the signaling machinery regulating maturation of the synaptic network. Nevertheless, regardless of the specific cellular mechanism, the notion that α2 subunit variants have epileptogenic potential is also supported by the observation that another mutation, Arg376Trp, is linked to the benign familial infantile seizure syndrome [85].

Further recent studies have extended our knowledge of the pathophysiology of α2. Genome-wide association studies point to *CHRNA2* as a risk locus in cannabis use disorder [86] and nicotine addiction [87], but not in schizophrenia [88] or bipolar disorder [89]. This is a further indication that a functional distinction can be traced between α2 and other nAChR subunits, whose loci are linked to the latter psychiatric disorders [90] and whose expression is altered in patients thereof [91,92]. Interestingly, nicotine addiction in an African American population [87] has been associated to α2^Thr22Ile^, one of the variants linked to ADSHE [80], suggesting a pleiotropic influence of abnormal α2 function. In conclusion, although evidence about the physiology and pathology of α2 nAChR is increasing at a relatively quick pace, the spectrum of its functions is still largely unknown and appears to be linked to heterogenous disease conditions, also involving peripheral tissue. An example is the *CHRNA2* association with overweight/obesity in a Korean population [93] and its implication in systemic energy homeostasis through a direct action onto adipocytes [94].

## 4. Other Genes Implicated in SHE

A list of identified or putative genes in ADSHE is given in Table 1. Further support to the notion that the cholinergic system is crucially implicated in SHE is given by the observation that a *recessive* form of the epilepsy is associated with mutant proline-rich membrane anchor 1 (PRIMA1), which anchors acetylcholinesterase (AChE) to the synaptic membrane [95]. AChE terminates the cholinergic signal by degrading the neurotransmitter. In the CNS, the functional form of AChE is associated as a tetramer with PRIMA1 on synaptic membranes, particularly to the proline-rich attachment domain located at the extracellular domain of PRIMA1 [96]. PRIMA1 is also implicated in intracellular processing and axon targeting of AChE. Defective AChE could lead to altered cholinergic responses, with an overstimulation of the nicotinic and muscarinic receptors [95]. Once again, however, the possible pathogenetic mechanism needs further clarification, as PRIMA deletion in mice indeed leads to higher ACh levels, but also to a high degree of phenotypic compensation [97].

Besides nAChRs, the other ion channel known to be implicated in ADSHE is the potassium sodium-activated channel subfamily T member 1 (K_Na_1.1, encoded by *KCNT1*). *KCNT1* is linked to severe forms of ADSHE [98] and other epilepsies, particularly epilepsy of infancy with migrating focal seizures [99,100], accompanied by neurological and psychiatric comorbidities [98,99,100]. The K_Na_1.1 mutations cluster around the cytoplasmic NADP^+^ binding domain of the channel [98] and lead to a “gain-of-function” phenotype in expression systems [100].

A major recent advance was the discovery that ADSHE can be caused by genes not encoding ion channels and not directly related to the cholinergic system (Table 1). In particular, mutant genes encoding proteins of the gap activity toward rags 1 (GATOR1) complex [101], such as the disheveled, egl-10 and pleckstrin domain-containing protein 5 (DEPDC5 [102,103,104,105,106]), and the nitrogen permease regulator-like-2 (NPRL2) and 3 (NPRL3, [107,108]) have been implicated in the pathogenesis of many focal epilepsies, including ADSHE. GATOR1 is a macromolecular complex that inhibits the mammalian target of rapamycin complex 1 (mTORC1, [101]), thus regulating the cellular sensing of nutrients levels and thereby brain homeostasis. Altered regulation of this pathway turns out to have a major impact on brain structure and function, causing a spectrum of monogenic neurologic diseases. Although the cellular mechanisms are still uncertain, hypoactivation of mTOR is implicated in focal epilepsy syndromes, whereas hyperactivation causes aberrant formation of neural circuit [101]. Altogether, these observations suggest that mTOR inhibitors could be added to the pharmacological toolkit in SHE, but clinical studies are lacking [109]. Other genes putatively implicated in ADSHE (Table 1) are the Ca^2+^-binding protein 4 [110] and the corticotropin-releasing hormone [111].

## 5. Steps toward Precision Medicine in ADSHE

Many common AEDs control neuronal hyperexcitability by targeting voltage- or ligand-gated ion channels. Although the antiepileptic action is frequently attributed to modulation of voltage-dependent Na^+^ channels (NaV), many channel blockers used to treat epilepsy or other paroxysmal disorders (e.g., cardiac arrhythmias) are rather promiscuous in their molecular targets [112,113]. A classic example is phenobarbital, which exerts its main action by increasing the mean open time of the GABA_A_ receptor (GABA_A_R) channel [114] but also blocks at similar concentrations voltage-gated Ca^2+^ channels (CaV), especially T-type (CaV_T_; [115]), and glutamate receptors (GluRs; [116]). Although the non-specific action of AEDs can widen the spectrum of unwanted side-effects, it may also constitute a first step towards precision medicine, i.e., by repurposing drugs that are particularly effective on the channel types known to be implicated in a given type of epilepsy. Such an approach has been attempted in *KCNT1*-related epilepsy, which is especially refractory to common AEDs. In vitro, the increased function of mutant K_Na_1.1 is fully reversed by quinidine [100], a well-known K^+^ channel blocker used as an antiarrhythmic and antimalaric. Unfortunately, clinical trials with quinidine on patients carrying *KCNT1* mutations gave mixed results, perhaps because of age-dependent effects and poor permeation through the blood–brain barrier [117,118,119]. Moreover, as other blockers of voltage-gated K^+^ channels (KV), quinidine is cardiotoxic, because of the tendency to facilitate fatal arrhythmias [118]. Nonetheless, it could constitute a lead compound to generate drugs with more specific molecular action and lower cardiotoxicity. A potentially useful molecular indication comes from the observation that quinidine is more effective on patients carrying mutations within the intracellular regulator of conductance of potassium 2 (RCK2) domain of K_Na_1.1, distal to the NADP^+^-binding site, implicated in the channel sensitivity to Na^+^ [119]. Another current strategy is to identify compounds that bind the channel pore more specifically than quinidine, by using computational methods based on the cryo-electron microscopy-derived K_Na_1.1 structure [120].

Carbamazepine is considered a first-line drug in SHE, although no extensive studies on wide cohorts of patients are available for other AEDs, and even carbamazepine is ineffective in >30% of patients [109]. This drug dampens neuronal firing by retarding the recovery from inactivation of NaV channels [121], but its action is not specific. In fact, the good efficacy of carbamazepine on ADSHE has been attributed to the fact that the drug also exerts open channel block of heteromeric nAChRs, with higher efficacy on some ADSHE-linked mutants [73,122,123]. Similar observations were carried out on other drugs found to be effective on SHE, such as oxcarbazepine [124,125], or on focal epilepsy in general, such as lamotrigine [126]. After absorption, oxcarbazepine is converted to 10,11-dihydro-10-hydroxy-carbamazepine (MHD). In humans, MHD is thought to be the therapeutic relevant compound, with plasma concentrations of 30 to 150 µM, as the steady state concentration of oxcarbazepine is negligible [127,128]. The spectrum of molecular targets of oxcarbazepine and MHD is poorly known. These compounds are thought to exert on NaV channels effects similar to those produced by carbamazepine [129], but other targets have been proposed. At the typical blood concentrations, MHD produces a ~40% open channel block of α4β2 nAChRs [123]. A similar reasoning applies to lamotrigine, which blocks α4β2 nAChRs in a range of concentrations (IC_50_ ~100 µM) overlapping with those effective on NaV channels [130].

Based on these findings, targeting nAChRs would appear to constitute a possible therapeutic method in ADSHE and offer the possibility of developing precision therapy in patients carrying different mutations. In principle, one can expect the effect of administration of nAChR agonists in vivo to be complex. The initial nAChR stimulation would be typically followed by channel desensitization, the extent of which would critically depend on the final drug concentrations and could vary among different mutant receptors. Probably because of its desensitizing effect, transdermal patches of nicotine were initially found to show anti-seizure efficacy in a patient carrying α4^Ser248Phe^ [131], and tobacco consumption was reported to be correlated with lower incidence of seizures in a group of patients displaying α4^Ser248Phe^ and α4^776ins3^ mutations [132]. A recent study carried out on three boys carrying α4^Ser248Phe^ (now designated as α4^Ser280Phe^) showed that treatment with nicotine led to drastic reduction of seizures and cognitive improvement [133]. Because of the rarity of these mutations, however, systematic evidence is still limited. Considering the encouraging results of the above studies, it would be important to test the effects of nicotine in wider cohorts of patients carrying different mutations on α4 as well as β2 nAChRs. We believe two critical aspects merit thorough investigation. First, nicotine treatment has also been found to be effective in patients not carrying nAChR mutations [134]. Therefore, it is essential to understand to what extent the antiseizure effects depend on general modulation of the frontal network or specific modulation of mutant nAChRs. Second, the long-term effects of nicotine depend on the compensatory response of the neocortex network to the drug. Tonic exposure to nicotinic agonists leads to upregulation of α4β2* nAChRs by different mechanisms, which include increased synthesis, membrane insertion, and stabilization of the expressed receptor [135]. These mechanisms are thought to cause the addictive effects of nicotine, especially through higher expression in the mesolimbic reward system [25]. It is clear that deeper studies are necessary to fully comprehend the balance between the potentially anti-seizure desensitizing effects of nicotinic agonists and the long-term action on receptors’ properties that could modulate the antiepileptic effect as well as cause unwanted cognitive and addictive side effects, particularly in adolescent patients.

A follow-up strategy would be to generate more specific compounds to target specific nAChR subtypes. With the currently available compounds, it is extremely difficult to distinguish effectively, e.g., α4* and α2* nAChRs (especially in vivo), which could nonetheless represent possible pharmacological targets for different forms of sleep-related epilepsy. A possible medicinal chemistry approach could be to focus on several known peptide toxins. For example, disulfide-deficient analogues of the αO-Conotoxin GeXIVA display higher affinity for α2β2 nAChRs compared to α7 and α3β4, although the comparison with α4β2 is still not available [136]. Unfortunately, from our perspective, most previous medicinal chemistry studies have focused on α7 receptors, because of their possible implication in neurodegenerative diseases [135]. From the point of view of strict precision medicine, the cost of planning the targeting of individual mutant channels would be prohibitive. Nonetheless, it should be possible to select compounds that allow to target different kinetic states, provided this information is available from studies in expression systems. Pursuing these studies should be considerably facilitated by the recent elucidation of high-resolution structure of heteromeric nAChRs [137]. A different approach would be to modulate the functional expression of nAChRs. For instance, nicotine itself was found to be able to normalize in vitro the balance of nAChR subunits in presence of ADSHE mutations which alter such balance [59]. Whether this strategy can be applied in vivo remains to be determined.

Alternatively, one could resort to compounds that regulate nAChRs less directly. For example, fenofibrate is effective in drug resistant SHE and ADSHE, when applied in addition to classic AEDs [138]. Fenofibrate is a clinically used agonist of peroxisome proliferator-activated receptor α, a transcription factor that when activated, is thought to negatively regulate β2-containing nAChRs, by phosphorylation mechanisms [138]. The AED effects discussed in the present Section are summarized in Table 2.

## 6. Developmental Aspects of ADSHE and Implications for Therapy

As is the case of other genetic epilepsies, the pathogenesis of ADSHE is increasingly recognized to present a developmental component [48,107]. This hypothesis was originally formulated after work in animal models conditionally expressing the β2^Val287Leu^ nAChR subunit [65]. Subsequently, the identification of *DEPDC5* loss of function mutations led to recognize a spectrum of epilepsy syndromes, among which ADSHE, associated with human brain malformation [101,106], as is also indicated by the first murine models in which *DEPD5* has been deleted [148,149,150].

Here, we limit our discussion to what is known about the possible developmental effects of mutant nAChRs in ADSHE. Several nAChR subunits (including β2) regulate synaptic maturation in the neocortex. In mice, expression of these subunits peaks between the 2nd and the 3rd postnatal week, a critical phase of synaptogenesis [84]. During this phase, α7 receptors are thought to regulate dendritogenesis and the maturation of glutamatergic synapses [151,152,153,154], whereas high-affinity β2* nAChRs, which may incorporate α5, are more specifically involved in the formation of dendritic spines and participate in the regulation of dendritic morphology [155,156,157,158]. A simple working hypothesis is that hyperfunctional nAChRs linked to sleep-related epilepsy could alter the Ca^2+^-dependent modulation of actin cytoskeleton that shapes spine structure and GluR distribution in excitatory synapses [152,156].

**Figure 1:**
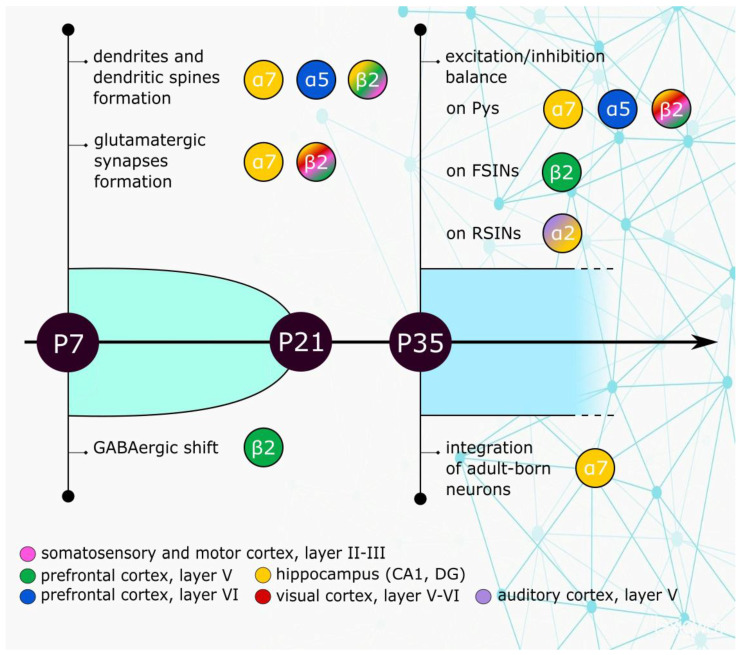
Implication of nicotinic acetylcholine receptor (nAChR) subunits at different postnatal stages. The indicated nAChR subunits regulate glutamatergic synapse formation and the GABAergic shift during the first 2–3 postnatal weeks. These notions mainly derive from experimental work in rodents [71,84,151,152,153,154,155,156,157,158]. After the first postnatal month, nAChRs assume their permanent function in cortical circuits, where they control the overall circuit excitability by regulating pyramidal neurons (Pys), fast-spiking GABAergic interneurons (FSINs), and other GABAergic populations (particularly somatostatin-expressing regular spiking non pyramidal cells, RSINs). The balance of nAChR function in pre- and post- (or extra-) synaptic sites in different layers and the kinetics of cholinergic effects are still matter of debate [23,24,29,30,31,32,33,41,42,43,48,54,76,163,164,165].

Another possible mechanism by which mutant nAChRs could affect synaptogenesis is suggested by the coincidence of the expression peak of nAChR subunits with the so-called “GABAergic shift” [159]. During early brain development, activation of GABA_A_Rs has a depolarizing effect which contributes to regulate brain morphogenesis. At later stages, GABA assumes the inhibitory role it normally has in the adult brain. Such a functional transition depends on the progressive decrease of neuronal intracellular chloride concentration ([Cl^−^]_i_). Because Cl^−^ is the main permeant ion in GABA_A_Rs, increasing the ratio between extra- and intracellular [Cl^−^] brings the reversal potential of GABA_A_ currents towards −70 mV, which allows the typical inhibitory action of GABA_A_Rs in mature networks. The [Cl^−^]_i_ decrease is brought about by the progressive increase of membrane expression of the Cl^−^ extruder K^+^-Cl^−^ cotransporter type 2 (KCC2), as compared to Na^+^-K^+^-Cl^−^ cotransporter 1 (NKCC1), which absorbs Cl^−^. In mice expressing β2^Val287Leu^, the KCC2 amount decreases around P8 in prefrontal cortex compared to the controls, thus delaying the GABAergic shift [71]. This is likely to be one of the contributing factors that cause the long-term synaptic alterations observed in ADSHE [48]. The main physiological roles of nAChR subunits in developing and mature cerebral cortex are summarized in Figure 1.

These notions point to the possibility of modifying the natural history of the disease, by pharmacological modulation of synaptic maturation, to obtain permanent beneficial effects. Studying how to target NKCC1 or KCC2 to regulate synaptogenesis is now a very active area of epileptology [159,160,161]. More specifically, in a rat model of ADSHE, the development of seizures was prevented by using furosemide, which can normalize Cl^−^ homeostasis by blocking NKCC1 [162]. The other major line of research that may lead to effective treatment during development is founded on the recent evidence about the developmental alterations produced by mutant components of GATOR1 (Section 4). Promising results have been obtained in a murine strain in which conditional deletion of *Depdc5* in dorsal telencephalic neuroprogenitor cells leads to macrocephaly, accompanied by spontaneous seizures and premature death. Early inhibition of mTORC1 with rapamycin improves survival and prevents seizures, which further encourages the search of effective developmental windows for anti-seizure treatment [150].

## 7. Conclusions

The possibility of carrying out sophisticated kinetic analyses of the effects of drugs targeting ion channels could lead to precision medicine aimed at modulating specific channel types and possibly individual mutations. In ADSHE, these studies can avail of the detailed 3D structural information now available for nAChRs. However, nAChRs as well as other ion channels have different functional roles in mature and developing brains. Therefore, future work should address the issue of the different effect of drugs targeting nAChRs or other molecules at different developmental stages. This is particularly important if one consider the very long brain maturation in humans. Pharmacological treatment could have different (even opposite) effects on synaptic maturation and adult excitability. Besides contributing to explain the variable effects produced by the same drug on different patients, these notions argue for the urgency of thorough epidemiological studies about the effects of different AEDs, from childhood to adult age. In addition, more work of a fundamental nature is needed to reach a general understanding of the long-term perturbations produced by single-site mutations in the adult and developing mammalian brain, whose nature is still largely obscure. We believe the different aspects of the treatment of ADSHE and other rare epilepsies are paradigmatic of the general problems encountered in understanding and curing epilepsy.

## Figures and Tables

**Table 1:** Identified or putative genes in autosomal dominant sleep-related hypermotor epilepsy (ADSHE).

Gene	Protein	Description	Clinical Phenotype	References
*CHRNA4*	α4 nAChR subunit	In heteromeric nAChRs	Typical SHE	[19,45,48,49,56]
*CHRNB2*	β2 nAChR subunit	In heteromeric nAChRs	Typical SHE	[46,47,48,56,58]
*CHRNA2*	α2 nAChR subunit	In heteromeric nAChRs	Seizures with nocturnal wandering and ictal fear	[72]
			SHE with paroxysmal arousals	[81,82]
*KCNT1*	K_Na_1.1 (also known as K_Ca_4.1,Slack, Slo2.2)	Na^+^-gated K^+^ channel	Severe SHE with psychiatric and cognitive alterations	[98]
			Epilepsy of infancy with migrating focal seizures	[99,100]
*DEPDC5* *NPRL2/3*	DEPDC5 Nitrogen permease regulator-like-2/3	DEPDC5 and NPRL associate to form GATOR1, which inhibits mTORC1	Wide spectrum of focal epilepsies, including SHE, often associated with brain malformation	[101,102,103,104,105,106,107,108]
*CABP4*	Ca^2+^-binding protein 4.	Regulates voltage-gated Ca^2+^ channels.	Typical SHE	[110]
*CRH*	Corticotropin-releasing hormone	Mutations in CRH promoter	Typical SHE	[111]

SHE: Sleep-related hypermotor epilepsy; nAChR: neuronal nicotinic acetylcholine receptor; DEPDC5: pleckstrin domain-containing protein 5; NPRL: nitrogen permease regulator-like; GATOR1: gap activity toward rags 1.

**Table 2:** Effects in vitro and in vivo of the antiepileptic drugs discussed in Section 5.

Compound	Ion Channel Targets	Effects on Ion Channels In Vitro	Efficacy on ADSHE-Linked Mutations In Vitro (Compared to WT)	Effects In Vivo (ADSHE/SHE Patients and Murine Models)	References
Carbamazepine	NaV	Delayed recovery from inactivation		First-line treatment for ADSHE. Ineffective in ~30% of the patients	[109,121]
	nAChRs	Open channel block of α4β2, α2β2, α2β4	Higher on α4^Ser248Phe^β2, α4^776ins3^β2, α2^Ile279Asn^β4; lower on α2^Ile279Asn^β2		[73,122,123]
	GABA_A_R	Potentiation of α1β3γ2 and α1β2γ2			[139,140]
	KV, CaV, GluRs.	Multiple effects overall leading to inhibition of glutamatergic transmission			[112,141,142]
Oxcarbazepine (metabolite of carbamazepine)				Effective on a fraction of patients insensitive to carbamazepine	[81,124,125]
	NaV	Inhibition. Negative shift of activation and inactivation of SCN9A ^1^			[129,143]
	nAChRs	Weak open channel block of α2β4			[123]
	GABA_A_R	Potentiation of α1β2 and γ2L subtypes	Higher efficacy on α2^Ile279Asn^β4		[144]
	Delayed rectifying KV.	Inhibition			[129]
	CaV	Inhibition; subtype specificity unknown			[112,142,145]
MHD (active metabolite of oxcarbazepine)				Overlaps with oxcarbazepine	[127,128]
	NaV and CaV	Inhibitory effects, but kinetic studies on specific subtypes are lacking			[112]
	nAChRs	Open channel block of α4β2; scarce effect on α2β4	N.D.		[123]
	GABA_A_R	No effect			[144]
Phenobarbital			N.A.	Reported to decrease seizures in patients with *KCNT1*-related Epilepsy	[146]
	GABA_A_R	Increases the mean open time			[114]
	CaV_T_	Block.			[115]
	GluRs	Block of GluR3 and GluR6			[116]
Quinidine				Mixed effects. Stronger antiseizure effects on patients carrying mutations of RCK2 domain of K_Na_1.1	[117,118,119]
	NaV	Use-dependent block			[147]
	K^+^ channels, included K_Na_1.1	Wide-spectrum K^+^ channel blocker	Reverses gain of function in mutant K_Na_1		[100]
Lamotrigine	NaV	Blocker			[113]
	nAChRs	Non-competitive inhibition of α4β2, included open channel block	N.D.	N.D.	[126]
Nicotine				Antiseizure effects on patients carrying α4^Ser248Phe^ or α4^776ins3^	[131,132,133]
	nAChRs	Activation, followed by desensitization	On mutant α4β2, often higher sensitivity to nicotine. (See main text)		[56,57,58,59,60,61]
				Increased sensitivity and induction of dystonic arousal complex in mice carrying α4^Ser248Phe^ or β2^Val287Leu^	[64,67]
Fenofibrate	nAChR (indirect effect)	Inhibits nAChRs by stimulating the peroxisome proliferator-activated receptor α (negative regulator of β2* nAChR)	In slices from mice carrying α4^Ser252Phe^: lower IPSC ^2^ frequency in cortical pyramidal neurons	Reduction of seizure frequency in drug-resistant SHE and ADSHE patients, if applied with classic AEDs	[138]

^1^ Sodium voltage-gated channel alpha subunit; ^2^ Inhibitory post-synaptic currents.
